# Adverse Events and Efficacy of Acetazolamide in Meniere’s Disease in a Vertigo Outpatient Clinic: A Retrospective Study

**DOI:** 10.7759/cureus.69616

**Published:** 2024-09-17

**Authors:** Teru Kamogashira, Shinnosuke Asakura, Hideaki Funayama, Shinichi Ishimoto

**Affiliations:** 1 Department of Otolaryngology, JR Tokyo General Hospital, Tokyo, JPN; 2 Department of Clinical Examination, JR Tokyo General Hospital, Tokyo, JPN

**Keywords:** acetazolamide, electrolyte imbalance, hypokalemia, liver dysfunction, meniere’s disease

## Abstract

Background

Acetazolamide, a carbonic anhydrase inhibitor, is used in the treatment of several otolaryngological conditions, including Meniere’s disease (MD). Despite its efficacy in reducing vertigo attacks, it is associated with a high rate of adverse events such as sensory disturbances, electrolyte imbalances, and liver dysfunction. The primary objective of this study is to evaluate the efficacy of acetazolamide in reducing vertigo attacks in MD patients. The secondary objective is to determine the incidence and severity of adverse drug events associated with acetazolamide treatment in this patient population.

Methodology

The study subjects were 70 patients who visited the vertigo outpatient clinic of our department from March 2019 to July 2024 and were diagnosed with definite or probable MD.

Results

A total of 15 cases had a history of acetazolamide prescription, with doses ranging from 125 to 750 mg/day. In total, 12 (80%) patients had symptoms of numbness in hands, feet, or other parts of the body, which caused discontinuation of medication after the initial prescription in five cases. Hypokalemia with liver dysfunction and hypokalemia with an open fracture of the distal end of the right radius due to staggering were observed in one patient each. In both cases of hypokalemia, Chinese herbal medicines containing licorice were prescribed in combination. Overall, 11 (73%) cases had a decrease in vertigo attacks. Hearing thresholds did not recover significantly after the prescription.

Conclusions

Although acetazolamide is effective in preventing vertigo attacks of MD, the rate of adverse events is high. Detailed instructions, careful dosage adjustment, periodic assessment of symptoms, and blood examinations for electrolyte and liver function are required.

## Introduction

Acetazolamide, a carbonic anhydrase inhibitor, has diuretic properties and is commonly prescribed for conditions such as sleep apnea [1], episodic ataxia [2-5], Meniere’s disease (MD) [6-9], and vestibular migraine [10]. In MD, acetazolamide is primarily used to manage vertigo attacks, but it is also associated with adverse effects, including numbness, paresthesia, electrolyte imbalances, and liver dysfunction [11,12]. The balance between therapeutic efficacy and potential adverse events is crucial for clinical decision-making. The primary objective of this study is to evaluate the efficacy of acetazolamide in reducing the frequency of vertigo attacks in patients with definite or probable MD. The secondary objective is to assess the incidence and severity of adverse drug events, particularly focusing on sensory abnormalities, electrolyte disturbances, and liver function.

## Materials and methods

This was a retrospective study conducted at a single institution. Patients referred to the vertigo outpatient clinic, Department of Otolaryngology at the JR Tokyo General Hospital from March 2019 to July 2024 and diagnosed with definite MD or probable MD based on the diagnostic criteria for MD by the Bárány Society [13] were included in this study. Data on acetazolamide prescription history, concomitant medications, adverse events, efficacy, scores of questionnaires regarding psychological symptoms, hearing thresholds, caloric testing results, and vestibular evoked myogenic potential (VEMP) results were collected. The prescription history was evaluated as of July 31, 2024. Because several cases discontinued medications after the initial acetazolamide prescription, efficacy was determined based on the reduction in subjective vertigo attacks after the end of the initial prescription. Questionnaires included the Dizziness Handicap Inventory (DHI) [14]; Self-rating Depression Scale (SDS) (age less than 65) [15] or Geriatric Depression Scale (GDS) (age 65 or over) [16,17]; Hospital Anxiety and Depression Scale (HADS) [18]; POUNDing (Pulsating, duration of 4-72 hOurs, Unilateral, Nausea, Disabling) [19]; Migraine Disability Assessment (MIDAS) [20] and four-item migraine screener (headache exacerbation in daily performance, nausea, light-sensitivity and hypersensitivity to odors) [21]; and screening checklist with major (five items) and minor (six items) manifestations for orthostatic dysregulation (OD score) [22].

Caloric testing was performed in a darkened room by irrigating the external auditory canal with 20 mL saline solution (4°C) for 10 seconds. Caloric nystagmus was recorded using videonystagmography (VNG, VN415, Interacoustics A/S, Middelfart, Denmark) and canal paresis percentage (CP%) was calculated from the maximum slow-phase velocity of nystagmus using Jongkees’ formula [23]. An abnormal caloric response was defined by either of the following criteria: (1) left or right canal paresis (CP); CP% ≥20% [24]; or (2) both side CP; maximum slow-phase eye velocity <10 degrees/second [25].

The cervical VEMP (cVEMP) and ocular VEMP (oVEMP) were recorded using the Nicolet EDX system (Natus) using a tone burst stimulus of 500 Hz at 125 dBpeSPL (rise, 1 ms; plateau, 2 ms; and fall, 1 ms) and a tone burst of 1 kHz at 125 dBpeSPL (rise, 1 ms; plateau, 2 ms; and fall, 1 ms). In cVEMP testing, electromyographic (EMG) activity was recorded from a surface electrode placed on the upper half of each sternocleidomastoid muscle (SCM), with a reference electrode on the side of the upper sternum and a ground electrode on the chin. During the recording, in the supine position, subjects were instructed to raise their heads from the pillow to contract the SCM. The EMG signal from the stimulated side was amplified and bandpass-filtered (20-2,000 Hz). The stimulation rate was 5.1 Hz, and the analysis time was 100 ms. Responses to 20 stimuli were an average of three times [26]. In oVEMP testing, subjects lay supine on a bed, with their head supported by a pillow and with surface EMG electrodes placed on the skin 1 cm below (active) and 3 cm below (indifferent) the center of each lower eyelid. The ground electrode was placed on the chin. During testing, the subject looked up approximately 30 degrees above straight ahead and maintained their focus on a small dot approximately 1 m from their eyes. The signals were amplified by a differential amplifier (bandwidth: 0.5-500 Hz). Responses to 20 stimuli were an average of three times [26]. An abnormal VEMP response was defined by the following criteria: asymmetry ratio (AR) percentage ≥33% or no response in both ears.

Changes in hearing thresholds before and after the prescription were compared between the thresholds on or before the start of prescriptions and the thresholds on the most recent day after the start of prescriptions. The averages of hearing thresholds of the three low frequencies (125, 250, 500 Hz) and the three middle frequencies (0.5, 1, 2 kHz) were evaluated.

Although this was a retrospective study and there is no standard protocol, the following methods were used. The basic dose of acetazolamide was 250 mg/day and was changed to 125 mg/day or 750 mg/day if the patient was underweight (<35 kg) or overweight (>75 kg). Patients were monitored for adverse events at each follow-up visit, which occurred every four weeks. Any symptoms of numbness, paresthesia, or other adverse effects were recorded. Electrolyte levels and liver function were tested at baseline, one month, and then every three months during treatment. Caloric testing, VEMP, and questionnaires were performed at baseline (before starting acetazolamide), and hearing threshold evaluations were performed at baseline and follow-up intervals of one to three months after the initiation of treatment.

For the statistical analysis of hearing thresholds, the paired t-test was performed using JMP 9 (SAS Institute Inc., Cary, NC, USA). P-values <0.05 were considered statistically significant.

This study protocol was reviewed and approved by the Research Ethics Committee of JR Tokyo General Hospital (approval number: R03-17). The information for this study was disclosed, and the participants could choose to opt out. An opt-out informed consent protocol was used for the use of participant data for research purposes. This consent procedure was reviewed and approved by the Research Ethics Committee of JR Tokyo General Hospital.

## Results

A total of 30 cases were diagnosed with definite MD and 40 were diagnosed with probable MD (Table 1). In total, 11 and 3 cases had a history of acetazolamide prescription in definite MD and probable MD cases, respectively. There were no cases in which acetazolamide was prescribed at the time of referral to the hospital. Acetazolamide was prescribed in cases with periodic vertigo attacks when side effects were explained and consent was obtained. There were no cases taking medications contraindicated with acetazolamide or cases with severe renal or hepatic impairment. The rate of prescription was higher in definite MD cases compared to probable MD cases. There were no cases of bilateral vestibular dysfunction. Of the 15 cases with acetazolamide prescription, 12 (80%) had symptoms of numbness in hands, feet, or other parts of the body; 11 (73%) had a decrease in vertigo attacks; 10 (67%) could continue taking the prescription at least twice; four (15%) were still continuing the prescription; and the maximum prescription duration was 1,099 days (Table 2). There were no cases of complete resolution of vertigo attacks. The prescribed doses ranged from 125 to 750 mg/day. Numbness was the most common reason for discontinuing prescriptions (five cases). Other adverse events included glossitis (Table 2, case 8), hypokalemia (K 2.9 mM), open fracture of the distal end of the right radius due to staggering (treated with open reduction and internal fixation) (Table 2, case 10), and liver dysfunction and hypokalemia (K 3.4 mM, aspartate transaminase 42 U/L, alanine transaminase 43 U/L, gamma-glutamyl transpeptidase 38 U/L) (Table 2, case 11). Concomitant medications were levothyroxine sodium, tofisopam, zolpidem tartrate, nicotinic acid amide/papaverine, diphenhydramine salicylate/diprophylline, and Kakkonto for case 10 and clotiazepam, esomeprazole, adenosine triphosphate disodium hydrate, mecobalamin, tranexamic acid, Hochuekkito, Daikenchuto, and Kakkonnto for case 11. Hypokalemia in case 10 was improved by discontinuation of the acetazolamide prescription and was treated with the oral administration of potassium asparagine, and liver dysfunction in case 11 was improved by discontinuation of the prescription. The prescriptions related to MD used before the start of acetazolamide prescription were isosorbide (13 cases), Goreisanryo (nine cases), Saireito (eight cases), and Hangebyakujutsu temmato (two cases) (Table 3).

**Table 1:** Case characteristics. Age, DHI, HADS, SDS, GDS, POUNDing, MIDAS, OD: average ± standard deviation. Statistical analysis was not performed due to the small number of cases. MD: Meniere’s disease; DHI: Dizziness Handicap Inventory; HADS: Hospital Anxiety and Depression Scale; SDS: Self-rating Depression Scale; GDS: Geriatric Depression Scale; POUNDing: Pulsating, duration of 4-72 hOurs, Unilateral, Nausea, Disabling; MIDAS: Migraine Disability Assessment; OD: orthostatic dysregulation; CP: canal paresis; VEMP: vestibular evoked myogenic potential; cVEMP: cervical VEMP; oVEMP: ocular VEMP; AR: asymmetry ratio

	With prescription	Without prescription
Total	15	55
Definite MD	11	19
Probable MD	4	36
Age	55.1 ± 12.5	58.0 ± 14.9
Age (median)	60	55
Sex (male)	53%	55%
DHI	36.4 ± 22.1	30.7 ± 22.3
HADS/A	6.1 ± 4.0	6.8 ± 3.8
HADS/D	6.0 ± 3.7	6.8 ± 4.0
SDS (age <65)	40.0 ± 9.5	43.0 ± 8.4
GDS (age ≧65)	0	4.0 ± 3.8
POUNDing	1.1±1.1	1.2 ± 1.3
Migraine screener (positive)	10%	16%
MIDAS	1.6 ± 1.0	1.2 ± 0.7
OD score	5.6 ± 3.4	4.8 ± 2.5
CP%	20.6 ± 14.2	20.3 ± 12.1
cVEMP (500 Hz) (AR ≧33%)	71%	63%
cVEMP (1 kHz) (AR ≧33%)	52%	54%
oVEMP (500 Hz) (AR ≧33%)	87%	69%
oVEMP (1 kHz) (AR ≧33%)	87%	64%

**Table 2:** Detailed information on each case. MD: Meniere’s disease; CP: canal paresis; AST: aspartate transaminase; ALT: alanine transaminase

#	Age	Sex	Diagnosis	LR	CP%	Dosage (mg/day)	Dosage (days)	Continuous prescription after the initial prescription	Stopped reason	Current prescription	Stopped reason	Effectiveness	Numbness					Electrolyte abnormality	Liver dysfunction	Fall down	Other symptoms and information
													Hand	Foot	Face	Neck	Unknown				
1	62	M	Definite MD	R	1	250	1	-	Numbness			-				+					
2	37	F	Definite MD	LR	10	125	21	-	Numbness			-	+	+	+						No skin sensation, ear fullness
3	59	M	Definite MD	L	19	750	21+28	-	Numbness			-					+				Re-prescription
4	53	F	Definite MD	R	14	250	3	-	Numbness			-					+				
5	60	M	Definite MD	LR	5	250	30	-	Improved			+	-								
6	53	M	Definite MD	L	6	250	196	+	-	-	Improved	+	+								
7	51	M	Definite MD	L	13	250	95	+	-	-	Improved	+	-								
8	61	F	Probable MD	L	29	250	31	+	-	-	Glossitis	+	-								Glossitis
9	60	F	Definite MD	R	41	250	28	+	-	-	Numbness	+	+	+							
10	78	F	Probable MD	R	20	250-750	161	+	-	-	Electrolyte abnormality	+	+					+		Staggering	Open fracture of distal end of right radius
11	63	F	Definite MD	R	45	250	674	+	-	-	Liver dysfunction	+	+					+	+		
12	38	M	Definite MD	R	2	250	77	+	-	+	-	+	+	+							Fatty liver (AST 31 U/L, ALT 61 U/L)
13	62	M	Definite MD	R	14	125	629	+	-	+	-	+	+								Clear eyes and head
14	61	F	Probable MD	R	38	250	957	+	-	+	-	+	+	+							
15	29	M	Definite MD	L	34	250	1099	+	-	+	-	+	+								

**Table 3:** Prescriptions before acetazolamide.

#	Prednisolone	Isosorbide	Goreisanryo	Saireito	Hangebyakujutsu temmato
1	〇	〇	〇	〇	
2	〇	〇			〇
3		〇	〇	〇	
4	〇	〇	〇	〇	
5	〇	〇	〇	〇	
6		〇		〇	
7				〇	
8		〇			
9		〇	〇		
10		〇			〇
11	〇	〇	〇		
12			〇	〇	
13		〇	〇		
14		〇			
15	〇	〇	〇	〇	

Hearing thresholds showed no significant changes after the acetazolamide prescription in the affected ear (the low average, p = 0.2; the middle average, p = 0.56). In the analysis of each of the left and right only, the left ear showed a significant threshold increase after the prescription (the low average of left, p = 0.76; the middle average of left, p = 0.03, the low average of right, p = 0.16; the middle average of right, p = 0.29).

**Table 4:** Hearing thresholds before and after prescription. Avg: average; low: the three low frequencies (125, 250, 500 Hz); mid: the three middle frequencies (0.5, 1, 2 kHz).

#	Inspection - prescription (days)	Before (left) (dB HL)									Before (right) (dB HL)									Prescription - inspection (days)	After (left) (dB HL)									After (right) (dB HL)								
		125	250	500	1000	2000	4000	8000	Avg Low	Avg Mid	125	250	500	1000	2000	4000	8000	Avg Low	Avg Mid		125	250	500	1000	2000	4000	8000	Avg Low	Avg Mid	125	250	500	1000	2000	4000	8000	Avg Low	Avg Mid
1	0	35	30	30	25	40	35	60	32	30	60	60	55	45	40	55	75	58	46	7	30	30	30	30	35	30	60	30	31	55	60	55	40	40	45	65	57	44
2	0	25	25	10	5	5	0	0	20	6.3	10	10	0	5	10	-5	0	6.7	5	6	30	35	25	5	5	0	-5	30	10	5	5	-5	5	5	-5	0	1.7	2.5
3	-70	65	75	75	75	65	70	80	72	73	20	25	30	40	45	55	65	25	39	63	70	90	85	85	70	65	75	82	81	15	20	30	40	45	55	60	22	39
4	0	20	15	10	0	0	0	15	15	2.5	45	45	45	25	5	15	20	45	25	62	15	10	5	5	0	0	15	10	3.8	25	25	15	-5	-5	0	10	22	0
5	-742	15	15	10	10	5	20	15	13	8.8	15	15	15	10	15	15	15	15	13																			
6	0	30	35	25	0	15	25	45	30	10	10	15	15	15	20	25	55	13	16	35	15	15	15	20	25	30	50	15	20	10	10	15	25	30	30	55	12	24
7	0	50	45	40	30	35	45	55	45	34	20	15	15	15	10	15	25	17	14	30	40	45	40	35	35	45	55	42	36	15	15	15	20	15	20	15	15	18
8	-133	15	15	10	5	10	25	20	13	7.5	10	10	10	0	10	25	25	10	5	77	20	15	15	10	10	25	15	17	11	20	20	15	5	15	20	55	18	10
9	0	15	10	5	10	10	25	65	10	8.8	60	60	60	60	55	50	55	60	59																			
10	0	15	15	10	5	20	20	65	13	10	45	45	45	40	25	25	60	45	38	35	10	10	10	5	20	20	60	10	10	40	45	40	35	30	30	60	42	35
11	0	20	20	10	5	15	5	30	17	8.8	55	55	50	25	25	15	30	53	31	105	20	15	10	5	20	5	40	15	10	35	30	15	15	25	15	40	27	18
12	-77	25	20	15	15	10	5	5	20	14	20	25	25	15	10	0	5	23	16																			
13	0	25	25	25	25	40	50	75	25	29	70	75	75	65	75	85	95	73	70	4	30	35	40	30	40	60	75	35	35	70	80	80	65	75	80	95	77	71
14	0	30	25	15	10	30	35	50	23	16	20	20	15	5	15	20	30	18	10	142	35	40	15	5	20	40	40	30	11	20	20	10	0	10	20	20	17	5
15	0	50	60	55	45	20	15	55	55	41	15	15	5	5	10	10	20	12	6.3	21	50	55	55	45	25	15	45	53	43	20	15	10	5	5	5	10	15	6.3

## Discussion

Adverse events of acetazolamide include headache, numbness, tinnitus, abdominal symptoms, electrolyte abnormalities (metabolic acidosis, hypokalemia, etc.), and Stevens-Johnson syndrome [12,27]. About half of the patients reported symptoms such as malaise, fatigue, weight loss, depression, anorexia, and decreased libido as adverse events of carbonic anhydrase inhibitors (acetazolamide or methazolamide) [28]. The frequency of adverse events of acetazolamide loading in quantitative cerebral blood flow scintigraphy was headache (14%), nausea (16%), peripheral numbness (38%), peripheral weakness (9%), and general malaise (24%), and 63% of patients had some kind of symptoms [12]. In glaucoma patients prescribed acetazolamide, unusual symptoms, including frequent urination, dry mouth, numbness, nausea, vomiting, and general malaise, were observed in 60% of patients [29], and adverse events, including frequent urination (26%), dry mouth (22%), abnormal feeling (17%), nausea (17%), and constipation (13%), were observed in 57% of patients [30]. A meta-analysis reported that the odds ratios of dysesthesia, dysgeusia, urinary frequency, and fatigue were 12.3, 4.2, 1.9, and 6.5, respectively, and the odds ratios tended to be higher in explicit symptom surveys [31]. There are few reports on the frequency of adverse events in vestibular disorders and the results of this study showed high frequencies of numbness; therefore, adverse events should also be carefully followed up in MD cases. Adverse events were the main reason for discontinuation after the initial prescription in this study. In a previous report evaluating the efficacy of acetazolamide for migraine, 35% of patients discontinued medication due to adverse events [32], so medication compliance should be continuously confirmed.

Prednisone is reported to be effective in treating MD [33] and is also prescribed in some cases for hearing loss [34]. Isosorbide [35], Goreisanryo [36], Saireito [37], and Hangebyakujutsu temmato [38] are all diuretics and are often prescribed for MD in Japan. The tendency for acetazolamide to be prescribed in definite MD may suggest that it is more difficult to treat than probable MD.

Severe hypokalemia occurred in the elderly in this study, suggesting that the elderly are susceptible to adverse events of acetazolamide. Although it has been reported that prescribing acetazolamide in addition to loop diuretics does not cause clinically significant hypokalemia [39], aging may affect the frequency of adverse events because pharmacokinetics of acetazolamide differs between elderly and young patients [40], and a decrease in renal clearance with aging [41] can also affect adverse events. The erythrocyte concentration and subsequent electrolyte abnormalities are important factors associated with the development of adverse events in patients receiving long-term acetazolamide treatment, indicating that unnecessary dosage increases of acetazolamide do not enhance efficacy [29]. Therefore, dosage increases of acetazolamide with long-term medication in elderly patients should be carefully evaluated. Although potassium supplementation has been reported to be ineffective in alleviating numbness [28], potassium supplementation may be useful in the treatment of hypokalemia due to the long-term administration of acetazolamide for glaucoma [42]. Further studies are needed to determine the efficacy of potassium supplementation in the continuous medication of acetazolamide in MD, including hypokalemia.

The concurrent use of other treatments, including diuretics and Chinese herbal medicines, may have contributed to some adverse events, such as hypokalemia, making it difficult to attribute these effects solely to acetazolamide. Concomitant medications used in all hypokalemia cases included Chinese herbal medicines containing licorice (Kakkonto and Hochuekkito), which is known to cause hypokalemia due to pseudohyperaldosteronism [43-45]. Hypokalemia improved after discontinuation of acetazolamide, suggesting that licorice was not the main cause of the electrolyte abnormalities in this study. However, concomitant use of licorice may contribute to exacerbation of electrolyte abnormalities because concomitant use of thiazide diuretics, loop diuretics, aldosterone antagonists, angiotensin-converting enzyme inhibitors, or angiotensin II receptor blockers may increase the risk of electrolyte abnormalities [46]. In addition, multiple Chinese herbal medicines are often prescribed for various symptoms and are self-adjusted according to conditions, so the total daily dose of licorice should be carefully evaluated [47]. Additionally, the variability in acetazolamide dosages across patients complicates the analysis, as it is unclear whether higher doses confer greater efficacy or simply increase the likelihood of adverse events. Future studies should aim to control for these confounders by standardizing treatment protocols and excluding patients on medications that may interact with acetazolamide.

Acetazolamide is sometimes prescribed for hyperkalemic periodic paralysis (HyperPP) [48] and hypokalemic periodic paralysis (HypoPP) [49], but it can exacerbate hypokalemia conversely [50]. Mutations in ion channel genes (HyperPP: *SCN4A*, HypoPP: *SCN4A*/*CACNA1S*) are the main cause of both diseases, and acetazolamide can reduce vertigo attacks to some extent in MD; hence, MD may be related to ion channel-related dysfunction [6]. Some previous studies have suggested the involvement of vasopressin receptor type 2 and aquaporin in MD [51-53], and the association of immunological disorders has also been studied [54]. In this study, there was no effect on hearing loss in the short term, and previous reports have also shown no long-term effect of acetazolamide on hearing loss [55]. The lack of significant changes in hearing thresholds suggests that while acetazolamide may reduce vertigo attacks, it may not play a significant role in improving or stabilizing hearing in patients with MD. Further studies should explore whether different treatment durations or dosages might have an impact on hearing outcomes.

One strength of this study is its detailed documentation of adverse events associated with acetazolamide in MD patients, providing valuable information on both the frequency and severity of these events. Additionally, the high efficacy rate observed in our patient population (73%) exceeds that reported in the previous study (46.2%) [56], suggesting that acetazolamide may be more beneficial in certain clinical contexts. This study adds important data on the balance between the efficacy and safety of acetazolamide in the management of MD, helping guide clinical decision-making.

However, this study has several limitations that should be noted. First, the retrospective design may introduce biases, particularly in the reporting of adverse events and the assessment of treatment efficacy. Patients with more severe symptoms or side effects may be more likely to discontinue treatment, potentially skewing the results. Additionally, the small sample size and the fact that this study was conducted at a single institution limit the generalizability of the findings. The use of concomitant medications, including Chinese herbal medicines and diuretics, further complicates the interpretation of adverse events, particularly electrolyte imbalances. Second, guidelines for evaluating its efficacy on vertigo attacks in MD [57] could not be utilized because prescriptions were discontinued in a large number of cases [58]. Lastly, the lack of long-term follow-up for all patients restricts our ability to assess the durability of the treatment effects and the long-term safety of acetazolamide.

Future research should focus on conducting multicenter studies with larger sample sizes to validate these findings in a broader population. Prospective studies would help reduce biases inherent in retrospective designs, particularly in tracking adverse events and treatment efficacy over time. Additionally, long-term follow-up studies are needed to assess the sustainability of vertigo reduction and investigate whether acetazolamide has any long-term effects on hearing preservation in MD patients.

## Conclusions

This study suggests that acetazolamide may be effective in reducing vertigo attacks in patients with MD; however, further large-scale, prospective studies are needed to validate these findings and determine the long-term safety of this treatment. The high rate of adverse events observed in this study, particularly electrolyte imbalances, may have been influenced by the concomitant use of medications such as Chinese herbal medicines containing licorice, which are known to affect potassium levels. This complicates the interpretation of the adverse effects solely as a result of acetazolamide.

Due to the retrospective design of this study, we cannot definitively establish a causal relationship between acetazolamide use and the reduction in vertigo attacks or the development of adverse events. Prospective studies with controlled conditions are needed to verify these associations. While acetazolamide appears effective in reducing vertigo attacks over the short term, the long-term efficacy and safety remain unclear due to limited follow-up in this study. Future research should focus on the long-term effects of acetazolamide use in MD. Careful explanation, careful dosage control, confirmation of concomitant medications, continuous checking of adverse events, and periodic evaluation of electrolyte and liver function should be considered in acetazolamide prescription.
